# Population Based National Tuberculosis Prevalence Survey among Adults (>15 Years) in Pakistan, 2010–2011

**DOI:** 10.1371/journal.pone.0148293

**Published:** 2016-02-10

**Authors:** Ejaz Qadeer, Razia Fatima, Aashifa Yaqoob, Sabira Tahseen, Mahboob Ul Haq, Abdul Ghafoor, Muhammad Asif, Masja Straetemans, Edine W. Tiemersma

**Affiliations:** 1 National Tuberculosis Control Program Pakistan, Islamabad, Pakistan; 2 KNCV Tuberculosis Foundation, The Hague, The Netherlands; Brighton and Sussex Medical School, UNITED KINGDOM

## Abstract

**Background:**

We aimed to determine the prevalence of pulmonary tuberculosis (TB) amongst the adult population in 2010–2011 in Pakistan.

**Method:**

A nationwide cross-sectional survey with multistage cluster sampling was conducted among adults (≥15 years) in 95 clusters in 2010–2011. All consenting participants were screened for cough and by chest X-ray. Participants with presumptive TB submitted two sputum samples for smear microscopy, culture, and molecular testing if needed. The TB prevalence estimates were adjusted for missing data and the cluster design.

**Result:**

Of 131,329 eligible individuals, 105,913 (81%) participated in the survey, of whom 10,471 (9.9%) were eligible for sputum examination. We found 341 bacteriologically positive TB cases of whom 233 had sputum smear-positive TB. The adjusted prevalence estimates for smear and bacteriologically positive TB were 270/100,000 (95% confidence interval (CI) 217–323), and 398/100,000 (95% CI 333–463), respectively. Only 61% of the diagnosed TB cases screened positive on symptoms (cough >2wks), whereas the other TB cases were detected based on X-ray abnormalities. The TB prevalence increased with age and was 1.8 times higher among men than women. The prevalence-to-notification ratio of smear-positive TB was 3.1 (95% CI 2.5–3.7), was higher among men than women, and increased with age.

**Conclusion:**

Our data suggest that there is under-detection and/or -notification of TB, especially among men and elderly. TB control should be strengthened specifically in these risk groups. X-ray examination should be combined with symptom screening to enhance case detection.

## Introduction

Tuberculosis (TB) is a global health problem. In 2014, an estimated 9.6 million people developed TB and 1.5 million died from the disease [1]. Currently, 22 high burden countries account for over 80% of world’s TB cases. Notification data in these countries often do not reflect the actual number of cases in the country due to incomplete coverage and absence of appropriate surveillance systems. Thus, direct measurement of the burden of disease through TB prevalence surveys remains key for understanding the spread and extent of the disease and aid in developing appropriate control measures in these settings [1,2].

In terms of absolute numbers of TB cases, in 2011, Pakistan ranked 5^th^ among 22 high burden countries. The estimated incidence and prevalence rates of all forms of TB were 231 (95% confidence interval (CI), 189–277) and 364 (95%CI, 154–611) per 100,000 population, respectively [3].

Effective implementation of the directly observed treatment, short course (DOTS) strategy recommended by the World Health Organization (WHO) started after revival of the National TB Control Program (NTP) in the year 2000, but was limited largely to the public sector services that fall under the responsibility of the NTP. The public sector has remained the main source of DOTS services since full DOTS coverage of the public sector was reached in 2005, but the estimated case detection rate remained low at 64%. A recent inventory study in Pakistan provided evidence that only 32% of diagnosed TB cases were notified to NTP, while the observed under-reporting of detected cases was 27% [4].

Prevalence estimates presented by the World Health Organization (WHO) for Pakistan were based on indirect estimation from notification data, assumptions about duration of TB disease, and expert opinion [3], because there was no recent nationally representative TB prevalence estimate available. The last TB prevalence survey was conducted in 1987–1989 [5], while since then, the TB epidemic in Pakistan has evolved and TB health services provision has improved [6]. To get a more precise understanding of the current TB burden, there was a need to conduct a new TB prevalence survey [7]. The primary objective of the survey was to estimate the prevalence of bacteriologically positive pulmonary TB amongst the adult population (≥15years) in a nationwide representative sample.

## Methodology

### Study setting and population

The estimated population size of Pakistan is about 182 million; approximately one-third being children (<15 years).The Gross Domestic Product (GDP) is increasing and was $4,500 in 2013 [8]. The health system of Pakistan is generally not strong and services are highly unregulated. Communicable diseases are still the leading cause of morbidity and mortality [9]; non-communicable diseases are on rise. While preventive care is mainly provided by the public sector, [10], the private sector is large and caters for about 75% of the population’s curative primary health care needs [11]. TB services are integrated into the primary health care system at district level and are delivered by public and private chest clinics in tertiary care, district and sub-district hospitals as well as rural health centres and basic health units. Through NTP, there is a vertical reporting, monitoring and supervision system in place.

The previous TB prevalence survey was conducted between 1987 and 1989 [5] and included symptom screening, chest X-ray and sputum smear microscopy. This third nationwide survey was stratified by province and by urban and rural areas, but the exact sampling strategy was not documented and remains unclear. In 41 clusters with approximately 989 individuals per cluster, 40,549 participants were included, resulting in a smear positive TB prevalence of 170/100,000 population. It is unclear how this estimate was obtained exactly.

The 4^th^ nationwide TB prevalence survey was conducted between August 2010 and December 2011. This was a cross sectional household-based survey using multistage cluster sampling following WHO guidance [2] conducted in 95 clusters with an intended sample size of 1,400 adults (aged > = 15 years) per cluster. The primary sampling unit consisted of *tehsils* (sub-districts).

A target sample size of 133,000 enumerated adults (> = 15 years) was calculated assuming an expected prevalence of smear-positive pulmonary TB of 213/100,000 population, a design effect of 2.5, a relative precision of 20%, and an expected participation rate of 85% [2]. Ninety-five clusters were sampled by probability proportional to the estimated *tehsil* population size in 2010 projected from 1998 census data (Federal Bureau of Statistics (FBS), 1998, unpublished data). A nationwide survey was planned but there was a need to exclude the following areas from the sampling frame because of serious security threats: the Federally Administrative Tribal Areas, district Dera-Bugti in Balochistan and 17 *tehsils* of Khyber Pakhtunkhwa, which together are inhabited by 6.4% of the country’s population (FBS, 2010, unpublished data). Five extra clusters were selected as backup, should the security situation in one of the 95 selected clusters deteriorate during the field work. The sample was not stratified.

Within each cluster, one union council (the lowest administrative division of Pakistan, UC) was randomly selected by using a random number table. During the fieldwork sketched maps of all selected UCs were obtained from the national Expanded Program for Immunization (EPI) to be able to select an area including 400 households; these micro plans were used for the random selection of one such cluster of households using a random number table. In rural areas, clusters of households usually consisted of one or more villages, while in urban areas two adjacent enumeration blocks of 200–260 houses within the jurisdiction of the selected UC were grouped to form one cluster of households.

### Measurements

In each cluster, the field work started with a door-to-door census in which all households within the selected area were visited and all eligible persons aged 15 years and above who slept in the household the night before the census were enumerated until the target number of 1,400 adults per cluster was reached.

Participants were those who took part in at least one of the screening methods. A screening interview was done at the reception desk, mainly by local lady health workers, by using a short symptom screening questionnaire. This questionnaire contained 5 questions and assessed current TB treatment status, presence of cough, cough duration, smoking status, and consent to X-ray screening. After the screening interview, all survey participants consenting to X-ray screening had chest X-ray examination, except pregnant women and those unable to have an X-ray because of physical disabilities. Chest X-ray images were read independently by two medical officers and discrepancies were solved by consensus. Those reporting cough for more than two weeks and/or with abnormal shadows on their chest X-ray image or reporting to be on TB treatment at the time of screening, and those with cough of any duration without (interpretable) chest X-ray image were considered presumptive TB cases.

### Laboratory examinations

Presumptive TB cases were subjected to an in-depth interview with questions on e.g. TB symptoms, and two sputum samples (spot and morning from the next day) were obtained. The spot specimen was examined in the field for the presence of acid fast bacilli (AFB) using direct smear microscopy with Ziehl-Neelsen staining. The morning specimen (or the spot specimen if no morning specimen was obtained) was transported in cold chain within 72 hours to the National reference laboratory for TB (NRL) in Islamabad for direct Ziehl-Neelsen smear microscopy and culture examination using the modified Kudoh method [12]. To identify *Mycobacterium tuberculosis* (MTB), all positive cultures were examined for morphology and subjected to paranitrobenzoic acid inhibition testing and/or MPB64 strip testing [13]. If needed, further identification was done using the Xpert MTB|RIF assay (Cepheid, Lyon, France). If culture results were not available while at least one smear was reported positive, a nucleic acid amplification test (NAAT), i.e., Genotype®MTBDR*plus* and/or Xpert MTB|RIF, was performed on scraped smear material.

### Quality assurance

All X-ray images with any abnormal shadows and 20% of X-ray images reported as normal were re-read by a central radiologist blinded to the field reading result. X-ray images with discordant result between field and central reading were reread by a senior radiologist for a final decision. All AFB-positive and 20% of the AFB-negative smears were reread at the NRL. In case any smear from a certain cluster was reported false-negative, all slides from that particular cluster were reread. The NRL results were considered as final in case of any discordance.

### Ethical issues

The survey started after obtaining ethical clearance from the National Bioethics Committee of the Pakistan Medical and Research Council. All procedures including consent and data collection were approved by NBC and adopted accordingly. Written informed consent with a signature or thumb print was obtained from all participants aged ≥15 years as this survey was confined to adults only. For some households in which the head of household considered obtaining informed consent from each individual household member as unnecessary or disrespectful, informed consent from the household head was considered sufficient for the complete household.

### Data management

All data forms and registers were sent to the central data management unit (DMU), where they were sorted, numbered and counted before filing.

For data entry, predefined data entry forms in Epi-Data version 3.1 (http://www.epidata.dk) were developed. A random sample of 10% of all forms and registers was double entered in a separate database and was compared to the original data entry. The discrepancies, occurring in <2% of all entries, were checked against the paper forms and corrected if necessary. Personal identification numbers (PINs) were checked and were corrected using information on cluster number, name, father’s name, age and sex.

### Definitions

A definite TB case was defined as having either an MTB-positive culture with five or more colonies; or less than five colonies in combination with a positive smear and/or an abnormal chest X-ray result consistent with TB; or a positive smear in combination with MTB confirmation on WHO-endorsed NAAT test.

A probable TB case was defined as having at least one positive smear in combination with an abnormal chest X-ray image but no MTB-positive culture (or NAAT result), nor non-*Mycobacterium tuberculosis* (NTM) grown on culture; or having two positive smears from two different specimens but no MTB-positive culture- or NAAT result nor NTM grown on culture.

A bacteriologically positive TB case was defined as any case with either definite or probable TB.

### Data analysis

Data was entered in EpiData version 3.1 and analysis was performed in STATA version SE 11.2 (STATA Corporation, College Station, TX, United States of America). P-values were calculated using chi-square tests and Cuzick test for trend where appropriate. A p-value of <0.05 was considered statistically significant. Prevalence estimates and 95%CIs for smear- and bacteriologically positive TB were calculated as recommended [14]. Prevalence estimates were obtained using two best practice methods described in detail elsewhere [14]: first by applying logistic regression with robust standard errors to account for the clustered design of the survey and second by adjusting for missing TB results among participants eligible for smear examination applying multiple missing value imputation methodology (using STATA ice and mi commands), as well as for non-participation by applying inverse probability weighting [14]. For these final estimates, 40 databases (each based on a 30-cycle iteration process) were imputed and combined to derive at a final adjusted prevalence estimate with 95%CIs. Sex and/or age were unknown for 6 participants, and these participants were not included in the analyses since imputation models failed to converge. None of these participants had presumptive TB. We calculated the prevalence-to-notification (P:N) ratio, which is currently being used as an indicator of program performance by the WHO [15].

## Results

**Fig 1:**
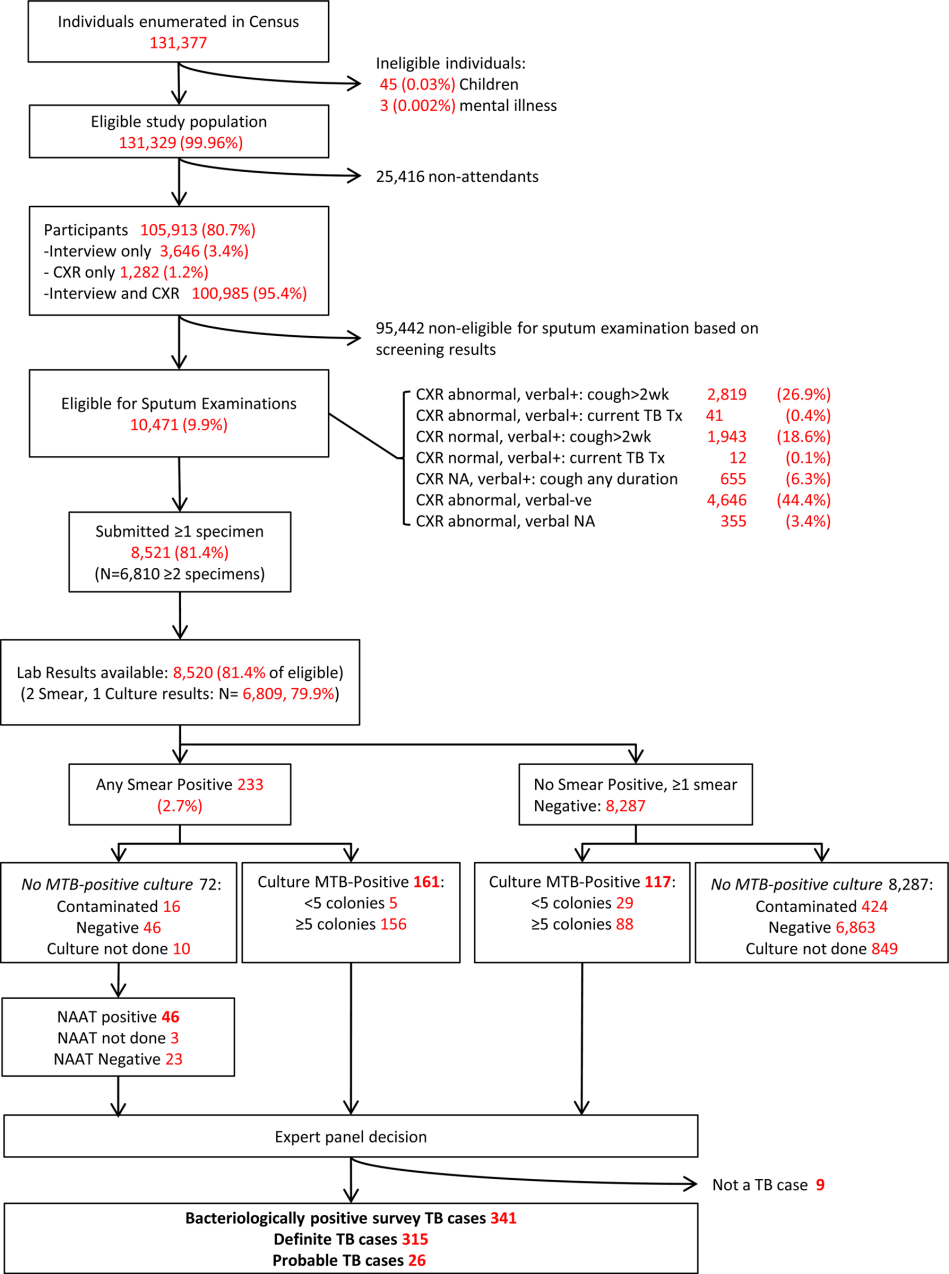
Flow Diagram of National Prevalence Survey of Pakistan, 2010–11.

In total, 131,377 persons were registered in the census, which was 98.8% of the target sample size. Of these, 131,329 were eligible, of whom 105,913 (80.6%) participated in the survey. Women were more likely to participate than men (88% versus 72%, p<0.0001). Although there were differences in participation rate over age groups and (geographical) areas, there were no clear trends visible (Table 1). Almost all (98.8%) participants were screened by questionnaire; 96.6% were screened by chest X-ray and 100,985 of these were screened using the questionnaire and chest X-ray (Fig 1).

**Table 1:** Characteristics of participants and non-participants to the national TB prevalence survey in Pakistan, 2010–2011.

Characteristic	Total eligible	Participated in at least one screening method					Among participants; screened by:			
		No		Yes		P-value	questionnaire:		X-ray:	
	N	N	%	N	%		N	%	N	%
Total	131,329	25,416	19.4	105,913	80.6		104,631	98.8	102,267	96.6
Sex						<0.0001				
Male	61,937	17,123	27.6	44,814	72.4		44,249	98.7	43,399	96.8
Female	69,388	8,289	11.9	61,099	88.1		60,382	98.8	58,868	96.4
Unknown*	4	4	100	0	0					
Age						<0.0001				
15–24y	41,920	8,227	19.6	33,693	80.4		33,368	99.0	32,521	96.5
25–34y	31,224	5,890	18.9	25,334	81.1		25,076	99.0	24,316	96.0
35–44y	24,531	4,823	19.7	19,708	80.3		19,476	98.8	19,041	96.6
45–54y	16,092	3,121	19.4	12,971	80.6		12,776	98.5	12,579	97.0
55–64y	9,161	1,733	18.9	7,428	81.1		7,288	98.1	7,247	97.6
65+ y	8,376	1,603	19.1	6,773	80.9		6,641	98.1	6,558	96.8
Unknown*	25	19	76	6	24		6	100.0	5	83.3
Province						<0.0001				
Punjab	79,342	15,207	19.2	64,135	80.8		63,425	98.9	61,761	96.3
Sindh	31,244	6,177	19.8	25,067	80.2		24,618	98.2	24,252	96.8
Balochistan	2,598	438	16.9	2,160	83.1		2,132	98.7	2,082	96.4
Azad-Jammu& Kashmir	4,321	979	22.7	3,342	77.3		3,318	99.3	3,280	98.1
Khyber Pakhtoon-Khwa	12,478	2,402	19.2	10,076	80.8		10,013	99.4	9,771	97.0
Gilgit-Baltistan	1,346	213	15.8	1,133	84.2		1,125	99.3	1,121	98.9
Type of area						0.02				
Rural	71,841	13,737	19.1	58,104	80.9		57,445	98.9	55,908	96.2
Urban	59,488	11,679	19.6	47,809	80.4		47,186	98.7	46,359	97.0

* Participants with unknown sex and/or age were excluded from all analyses for calculation of prevalence estimates.

Of 104,631 persons screened on symptoms, 5,470 (5.2%) qualified for sputum examination (Table 2). Among these, 5,063 (92.6%) complained of cough for more than 2 weeks, 354 (6.5%) had cough for a shorter or unknown duration and were not on TB treatment, but had no chest X-ray result available, and 146 (2.7%) were listed as being most likely on TB treatment at the time of the survey. Men more often complained of prolonged cough than women (p<0.001), and the prevalence of cough of more than 2 weeks increased with age (p<0.001, Table 2).

**Table 2:** Results of screening by questionnaire and participant characteristics from the national TB prevalence survey in Pakistan, 2010–2011.

Characteristic	Total screened*	Current cough >2 weeks		Current cough< = 2 wks, no X-ray**		current TB treatment		Any of these	
	N	N	%	N	%	N	%	N	%
**Total**	104,631	5,063	4.8	354	0.3	146	0.14	5,470	5.2
**Sex**									
Male	44,249	2,343	5.3	135	0.3	74	0.17	2,498	5.6
Female	60,382	2,720	4.5	219	0.4	72	0.12	2,972	4.9
**Age**									
15–24y	33,368	817	2.4	95	0.3	35	0.10	928	2.8
25–34y	25,076	799	3.2	87	0.3	26	0.10	899	3.6
35–44y	19,476	908	4.7	76	0.4	25	0.13	992	5.1
45–54y	12,776	896	7.0	49	0.4	18	0.14	951	7.4
55–64y	7,288	743	10.2	22	0.3	22	0.30	768	10.5
65+ y	6,641	900	13.6	25	0.4	20	0.30	932	14.0
**Province**									
PUNJAB	63,425	3,342	5.3	228	0.4	67	0.11	3,592	5.7
SINDH	24,618	1,061	4.3	66	0.3	36	0.15	1,136	4.6
BALOCHISTAN	2,132	45	2.1	16	0.8	7	0.33	64	3.0
AJK	3,318	143	4.3	6	0.2	18	0.54	158	4.8
KPK	10,013	445	4.4	36	0.4	15	0.15	488	4.9
GILGIT-BALTISTAN	1,125	27	2.4	2	0.2	3	0.27	32	2.8
**Type of cluster**									
Rural	57,445	3,243	5.6	238	0.4	97	0.17	3,513	6.1
Urban	47,186	1,820	3.9	116	0.2	49	0.10	1,957	4.1

* of total number screened by questionnaire.

**This category only includes participants who had cough but for a period of less than or equal to 14 days AND were not on TB treatment at the time of the survey AND for whom no X-ray result was available.

Chest X-ray images were taken from 102,267 participants of whom 7,828 persons (7.7%) had any abnormality on their X-ray image (Table 3). The proportion of men with an abnormal X-ray image was slightly higher than the proportion of women (p = 0.001). The proportion of X-ray abnormalities increased with age (p<0.0001; Table 3).

**Table 3:** Results of field X-ray screening by participant characteristics of participants to the national TB prevalence survey in Pakistan, 2010–2011.

**Characteristic**	**Total screened on X-ray**	**Field X-ray result**					
		**Normal**		**Abnormal**		Not available*	

	N	N	%	N	%	N	%
**Total**	102,267	93,424	91.4	7,828	7.7	1,015	1.0
**Sex**							
Male	43,399	39,474	91.0	3,491	8.0	434	1.0
Female	58,868	53,950	91.7	4,337	7.4	581	1.0
**Age**							
15–24y	32,521	31,303	96.3	909	2.8	309	1.0
25–34y	24,316	23,002	94.6	1,047	4.3	267	1.1
35–44y	19,041	17,375	91.3	1,486	7.8	180	1.0
45–54y	12,579	11,021	87.7	1,442	11.5	116	0.9
55–64y	7,247	5,870	81.1	1,306	18.0	71	1.0
65+ y	6,558	4,848	74.0	1,638	25.0	72	1.1
Unknown	5	5	100.0	0	0.0	0	0.0
**Province**							
PUNJAB	61,761	56,098	90.9	4,968	8.1	695	1.1
SINDH	24,252	22,207	91.6	1,811	7.5	234	1.1
BALOCHISTAN	2,082	1,971	94.8	97	4.7	14	0.7
AJK	3,280	3,065	93.4	195	5.9	20	0.6
KPK	9,771	9,043	92.6	681	7.0	47	0.5
GILGIT-BALTISTAN	1,121	1,040	92.8	76	6.8	5	0.5
**Type of cluster**							
Rural	55,908	50,420	90.2	4,759	8.5	729	1.3
Urban	46,359	43,004	92.8	3,069	6.6	286	0.6

*Empty form available (result not filled on form for n = 933), or no form but an X-ray image available (for n = 82).

In total, 10,471 participants (9.9%) were eligible for sputum examination, of whom 8,521 (81.4%) submitted at least one sputum specimen for bacteriologic diagnosis of TB (Fig 1). AFB smear results were obtained on two specimens for 6,809 persons and on one specimen for 1,711 persons. Of 7,661 samples cultured, 7,221 (94.3%) yielded an interpretable (positive/negative) culture result.

The survey identified 233 smear-positive TB cases; 161 of these were positive on culture while for 46 cases with no culture results available, MTB was confirmed on NAAT. Twenty-six smear-positive cases (including 19 with a single positive smear and 7 with two positive smears) were not confirmed on culture nor molecular testing. These 26 cases were listed as probable TB cases. There were 108 smear-negative definite TB cases; 88 of these had ≥5 colonies on culture. In total, 341 TB cases were identified in the survey (Table 4).

**Table 4:** TB cases according to case definitions.

TB case definition			N	Total N
**Probable TB**		2 SS+ no cult/ NAAT confirmation	7	26
		1 SS+, abnormal X-ray no C/NAAT confirmation	19	
**Definite TB**	**SS+**	C+ 5+ colonies	156	315
		C+<5col & (SS+ or abnormal CXR)	5	
		NAAT+ and SS+	46	
	**SS-**	C+ 5+ colonies	88	
		C+<5col & (SS+ or abnormal CXR)	20	
**Total**				341

SS+ = Sputum smear positive, NAAT = Nucleic Acid Amplification Test, C+ = Culture positive, col = Colonies, CXR = Chest X-ray, C+<5col & (SS+ or abnormal CXR) = Sputum smear and culture positive with <5colonoies but abnormal X-ray.

Of 207 definite smear-positive TB cases, 49% had cough for more than 2 weeks and X-ray abnormalities suggestive for TB, 39% had no cough but an abnormal chest X-ray image, and 12% had only cough for more than 2 weeks, but a normal chest X-ray image. Only 7.6% of the 341 participants with bacteriologically positive TB reported to be currently on TB treatment.

The final prevalence of smear and bacteriologically positive TB was respectively 270.3 (95% CI 217.3–323.3) and 397.9 (95% CI 333.2–462.6) per 100,000 population. The prevalence of bacteriologically positive TB was 1.5 times higher among men than women (p = 0.001) and 1.5 times higher in rural compared to urban areas (p = 0.009); it significantly increased with age and was highest in Sindh province (Table 5). Sensitivity analyses assume that probable TB cases with a negative NAAT result (n = 23) had no TB, or no conclusion regarding pulmonary TB could be reached for the probable TB cases (n = 26) with slightly lower estimates of 370 and 371/100,000 respectively, within the confidence intervals of the estimates presented in Table 5.

**Table 5:** Estimated prevalence of sputum smear-positive and bacteriologically positive TB among adults (≥15 years) in the national Pakistan prevalence survey, 2010–2011.

	Sputum smear-positive TB								Bacteriologically positive TB						
Characteristic	N cases	Unadjusted TB prevalence*	95 CI		Adjusted TB prevalence**	95 CI		N cases	Unadjusted TB prevalence*	95 CI		Adjusted TB prevalence**	95 CI	
**Total**	233	224.1	179.9	268.4	270.3	217.3	323.3	341	328.0	275.2	380.8	397.9	333.2	462.6
**Sex**														
Male	144	304.6	237.7	371.5	351.9	272.9	430.9	181	411.4	334.4	488.4	484.4	392.2	576.5
Female	99	165.1	123.8	206.4	197.2	145.0	249.4	160	266.8	213.6	320.0	320.4	252.6	388.3
**Age**														
15–24y	45	135.4	92.3	178.5	179.5	120.1	238.8	60	180.5	130.9	230.1	241.6	168.4	314.9
25–34y	31	124.3	78.3	170.4	163.0	100.2	225.8	42	168.4	110.1	226.7	228.0	149.0	307.1
35–44y	46	238.3	161.4	315.2	293.1	195.5	390.7	61	316.0	219.3	412.7	397.9	274.9	520.8
45–54y	42	331.8	222.4	441.2	391.9	253.9	529.8	55	434.5	311.3	557.7	516.6	362.2	671.0
55–64y	26	359.4	217.6	501.1	385.5	230.6	540.4	39	539.0	349.6	728.5	587.0	377.3	796.6
65+ y	43	653.2	425.6	880.8	690.5	438.6	942.4	84	1276.0	970.0	1582.1	1369.1	1028.3	1709.8
**Province**														
Punjab	137	217.7	166.0	269.4	262.5	192.6	332.5	199	316.2	255.7	376.7	378.8	287.4	470.3
Sindh	69	281.5	161.1	401.8	345.0	193.5	496.5	89	363.1	229.0	497.1	454.9	272.3	637.5
Balochistan	3	142.0	88.5	195.5	174.7	77.6	271.9	4	189.3	172.7	205.9	279.9	69.3	490.6
Azad-J. & Kashmir	6	181.8	92.8	270.9	203.4	107.5	299.2	10	303.0	159.0	447.1	381.6	138.2	625.1
KhyberPakhtoon-Khwa	16	160.3	49.6	271.0	177.5	49.4	305.6	37	370.7	169.2	572.1	417.9	173.6	662.2
Gilgit-Baltistan	2	179.1	179.1	179.1	205.7	104.1	307.3	2	179.1	179.0	179.1	239.9	76.4	403.4
**Type of area**														
Rural	155	272.5	204.5	340.4	321.1	241.3	400.9	224	393.7	316.0	471.4	470.5	377.1	563.9
Urban	78	165.7	118.7	212.8	208.6	146.9	270.4	117	248.6	187.9	309.2	310.0	234.4	385.6

* Including participants with full information only, with confidence intervals based on robust standard errors.

**Adjusted for missing information on outcome (TB disease Y/N) among participants using multiple imputation and adjusted for missing information among all eligible persons using inverse probability weighting, described as Method 3 by Floyd et al[14].

The ratio of the prevalence of smear-positive TB per 100,000 population to the notification rate of new smear-positive TB per 100,000 persons for 2011 (P:N ratio) was 3.1 (95% CI: 2.5–3.7) overall (Table 6). It was significantly higher among men than women (p = 0.003) and among those aged 65 years and above compared to other age groups (p<0.0001). The P:N ratio was highest in Gilgit-Baltistan and lowest in Khyber Pakhtoon-khwa (p<0.0001).

**Table 6:** Estimated prevalence-to-notification ratios, comparing the estimated prevalence of smear-positive TB with the notification of new-smear positive TB for the Pakistan National Tuberculosis Program, by sex, age group and province, 2011.

Characteristic	Adjusted* TB prevalence per 100,000 population from the prevalence survey	TB case notification rate per 100,000 population in 2011	Prevalence-to-notification ratio (P:N)	95 CI (P:N)	
				Low	High
		
Total	270.3	86.5	3.1	2.5	3.7
**Sex**					
Male	351.9	89.0	4.0	3.0	4.8
Female	197.2	83.8	2.4	1.7	2.9
**Age group**					
15–24y	179.5	70.7	2.6	1.7	3.3
25–34y	163.0	63.0	2.6	1.6	3.6
35–44y	293.1	84.3	3.5	2.3	4.5
45–54y	391.9	150.4	2.6	1.7	3.6
55–64y	385.5	135.5	2.9	1.7	4
65+ y	690.5	131.1	5.3	3.3	7.1
**Province/Region**					
Azad-Jammu and Kashmir	203.4	51.6	4.0	2.1	5.9
Balochistan	174.7	51.8	3.3	1.5	5.3
Gilgit-Baltistan	205.7	25.0	8.3	4.2	12.5
Khyber Pakhtoon khwa	177.5	84.2	2.1	0.6	3.6
Punjab	262.5	92.7	2.9	2.1	3.6
Sindh	345.0	85.7	4.0	2.3	5.9

* The prevalence of smear-positive TB was estimated from the prevalence survey, and adjusted for missing information on outcome (TB disease Y/N) among participants using multiple imputation and adjusted for missing information among all eligible persons using inverse probability weighting, described as Method 3 by Floyd et al[14].

Note: these figures were not age-adjusted.

## Discussion

This TB prevalence survey among adults (≥ 15 years) was the second largest prevalence survey ever, after China [16]. A total of 8,521 sputum smears were examined, over 7,500 cultures were performed and 341 TB cases (315 definite and 26 probable cases) were identified.

The prevalence of sputum smear positive TB in the survey was 270 per 100,000 population (95% CI 217–323), whereas the previous national TB prevalence survey in Pakistan conducted in 1987–1989 provided TB prevalence estimate of 170/100,000 population [5]. However, it is difficult to compare results of these two surveys, as for the previous survey the exact sampling design and methodology used for screening and diagnosis of TB, as well as the age and sex distribution of TB cases, remain unknown. The 2010–2011 survey used more sensitive chest X-ray screening methodology than the 1987–1989 survey (full size digital X-ray *versus* mass miniature radiography (MMR)). Other possible explanations for the higher prevalence in the 2010–2011 survey include the increased economic and political instability since 1987 which may have led to more inequity in access to health care (about 80% of the population having none) [17] and several individual level predisposing factors for TB of which the prevalence increased in the past decade, such as under nutrition, indoor air pollution, smoking, and diabetes [18].

We report a prevalence of bacteriologically positive TB (≥15 years) of 398 per 100,000 population (95% CI 333–463). Using this estimate and applying assumptions about the prevalence of extra-pulmonary TB and the prevalence of TB in children, the WHO obtained a prevalence estimate for all forms of TB among all age groups of 342/100,000 (95% CI 284–406), adjusted for the likely underestimation of the prevalence of bacteriologically positive TB due to applying culture on only one instead of two sputum specimens [19]. The TB prevalence estimate is similar to the prevalence estimate published by the WHO in 2011 (364 (95% CI 154–611) per 100,000 population), but with a much narrower confidence interval [3].

We faced several challenges in the conduct of the survey. Lack of understanding of the importance of PINs by the local survey staff (lady health workers) led to errors in assigning PINs to participants at the registration booth. Initially, around 8% of the participants’ PINs were not available in the census register. All survey PINs (even if there were no indications for wrong merges) were checked using name and father’s/husband’s name, and wrong PINs and merges were corrected. Ultimately <1% of participants were not found in the census; these participants were excluded from the survey. Four of these were diagnosed with bacteriologically positive TB (two probable and two definite cases).

Initially, cluster visits were scheduled in the morning. This led to very low participation (54% in the first pilot cluster), especially among men. Therefore, we expanded the opening hours of the cluster field sites to also include evenings. However, the participation rate remained relatively low (80.6%) compared to most, but not all, other Asian TB prevalence surveys [15]. Stigma may partially explain the low participation rate, especially in rural areas. Other potential explanation include higher socio-economic status in urban areas. The sputum collection rate was low compared to other surveys (81.4%) [15]. Reasons for this low rate are unclear.

In this survey, smear positive cases accounted for 68% of all bacteriologically positive cases and this proportion was relatively high compared to others recent surveys [15,16,20–23]. This may be due to the fact that only one specimen was subjected to culture and this strategy tends to underestimate the prevalence of bacteriologically positive TB [24]. The Vietnam survey also applied one instead of two cultures, and the proportion of smear positive cases was comparable at 65% [25]. The surveys in Cambodia and Lao included two cultures and the proportions of smear positive cases were 33% and 45% respectively [21,23]. The fact that the majority of the smear-positive, culture negative samples (32/46) was confirmed to be MTB-positive using NAAT suggests that applying one instead of two solid cultures leads to underestimation of the prevalence of smear-negative, culture positive TB. The low culture recovery rate may be due to relatively long sample transportation times under harsh climate conditions. Contrarily, we may have overestimated the prevalence of smear-positive TB: 23 of the 26 probable TB cases were not confirmed using NAAT directly on sputum smears. This may be explained by the low sensitivity of the Genotype®MTBDR*plus* test when applied directly on sputum smears, but it is also possible that these AFB-positive smears contained NTM instead of *M*. *tuberculosis* strains, as was found in some recent surveys [15]. However, only 3% of the positive cultures contained NTM (and no *M*. *tuberculosis*). Sensitivity analyses, assuming that the 23 probable TB cases with a negative NAAT result had in reality no TB, or assuming that no conclusion regarding pulmonary TB could be reached for the 26 probable TB cases, yielded slightly lower prevalence estimates which were in range with the estimates presented in this paper.

The prevalence of bacteriologically positive TB was 1.5 times higher and the P:N ratio was 1.7 times higher in men than women. A higher prevalence of TB among men has been reported from almost all other countries in the world [1,15,26], and other TB prevalence surveys in Asia have shown higher P:N ratios for men than women [15]. This suggests a higher TB burden among men combined with poorer health care seeking behaviour. Possible explanations for the higher TB burden in men are that men generally have more interaction with people outside their own home, are more often smoking, and are more exposed to risk factors during both work and leisure time[27,28].

The TB prevalence increased with age; a similar pattern is also observed in the TB surveillance data of Pakistan. The higher TB prevalence among elderly population may be explained by the recurrence of TB from endogenous reactivation in combination with a weak immune system rather than recent transmission [29,30]. It should be noted however, that this trend by age was much less pronounced than in other published Asian TB prevalence surveys [15], suggesting that active transmission of TB still occurs at high rates.

The prevalence of bacteriologically positive TB was higher among rural than urban residents (adjusted prevalence, 471 vs. 309, p<0.0001), while TB is usually thought of as a disease of urbanization [31]. Although this effect is largely explained by the different age distribution (mean age in rural *versus* urban areas 35.4 (SD, 23.0) *versus* 33.6 (17.6) years, p<0.0001), the higher TB prevalence in rural areas is probably also partially the result of longer disease duration, because of limited availability of health services and the longer distances to health facilities in combination with poverty and poor knowledge and awareness about TB [32–34].

The TB prevalence was highest in Sindh province which is one of the most populous provinces in Pakistan. Azad-Jammu and Kashmir, Balochistan, Gilgit-Baltistan and Khyber Pakhtoon-khwa have a lower population density, and higher elevation levels, which may both be protective for transmission of TB infection. Higher altitudes are related to more exposure to sunlight and higher vitamin D levels, which may protect against progression to TB disease [35].

TB notification in a country is expected to be similar to the TB incidence if there is a well-functioning NTP surveillance system and almost all cases are notified. We report a P:N ratio of 3.1, and a low proportion of TB cases being currently on TB treatment (7.6%). This suggests significant under-detection and under-reporting of TB in Pakistan. This was also the conclusion of a recent study conducted in 12 districts across Pakistan, which estimated the proportion of cases notified to NTP to be 32% [4]. The private sector is huge in Pakistan and less than 1% of the private providers are reporting TB cases to NTP [36]. The P:N ratio was higher among males compared to females, among elderly compared to other age groups, and among the population of Gilgit-Baltistan compared to the population of other provinces, suggesting lower case detection and/or notification in these subpopulations. This suggests that NTP should accelerate its efforts of focusing on these subpopulations to increase TB case detection and/or improve case notification. Another reason for low TB case detection may be that the National guideline prescribes that only clients complaining of cough existing for more than 2 weeks are screened for TB, and this guidance is generally followed in routine settings. This screening algorithm is rather insensitive as shown by our data: only 61% of the TB cases diagnosed in the survey screened positive on symptoms (cough >2wks), whereas the other cases had sputum examined based on X-ray abnormalities.

We corrected for missed TB cases among non-participants by applying inverse probability weighting and by missing value imputation for participants with missing (laboratory) results. However, this approach assumes that data are missing at random [37] whereas in fact, other unmeasured factors (such as job type, socio-economic status, stigma, etc.) may have influenced participation and it is thus possible that we could not fully adjust for missing values due to non- or partial participation.

The survey was limited to areas that were safe enough to visit and we had to exclude 6.5% of the Pakistan population from the sampling frame. As most of these Northern and Western areas are scarcely populated, we expect that these areas have relatively low TB prevalence rates. Hence, the exclusion of these areas may have led to a slight overestimation of the TB prevalence estimate in our survey [6]. As all published national TB prevalence surveys, we excluded the military, police, prisoners, persons living in internally displaced persons camps, and homeless people. The effect of exclusion of these groups on the TB prevalence remains unknown. Also, this survey does not provide information on extra-pulmonary TB cases, which constitute 17% of all notified cases in Pakistan [6], and it was limited to adults, because the diagnosis of (pulmonary) TB is difficult among children. We did not measure HIV-status. However, the HIV-prevalence among the general population in Pakistan is known to be stable and very low, and confined to certain high-risk groups [38].

The results of this national TB prevalence survey among adults (≥ 15 years) suggest that the bacteriologically positive TB prevalence is high and TB remains a public health problem in Pakistan. Efforts should be made to increase TB case detection, especially among men and elderly, especially in Azad-Jammu and Kashmir, Sindh and Gilgit-Baltistan, and to improve the TB surveillance system so that most TB cases will be notified to NTP in the future. To achieve this, there is a need to strengthen TB case detection, including introduction of innovative case finding techniques and strategies, as well as to enhance TB case detection and notification by involving private providers.
